# Next generation sequencing of carcinoma of unknown primary reveals novel combinatorial strategies in a heterogeneous mutational landscape

**DOI:** 10.18632/oncoscience.352

**Published:** 2017-06-23

**Authors:** Ishwaria M. Subbiah, Apostolia Tsimberidou, Vivek Subbiah, Filip Janku, Sinchita Roy-Chowdhuri, David S. Hong

**Affiliations:** ^1^ Department of Palliative, Rehabilitation, and Integrative Medicine, University of Texas MD Anderson Cancer Center, Houston, TX, USA; ^2^ Department of Investigational Cancer Therapeutics, Division of Cancer Medicine, University of Texas MD Anderson Cancer Center, Houston, TX, USA; ^3^ Department of Pathology, all at the University of Texas MD Anderson Cancer Center, Houston, TX, USA

**Keywords:** carcinoma of unknown primary, next generation sequencing

## Abstract

**Background:**

Advanced carcinoma of unknown primary (CUP) has limited effective therapeutic options given the phenotypic and genotypic diversity. To identify future novel therapeutic strategies we conducted an exploratory analysis of next-generation sequencing (NGS) of relapsed, refractory CUP.

**Methods:**

We identified patients in our phase I clinic where archival tissue was available for a targeted NGS CLIA-certified assay.

**Results:**

Of 17 patients tested, 15 (88%) demonstrated genomic alterations (median 2 aberrations; range 0–8, total 59 alterations). Nine (53%) patients had altered cell signaling including the PI3K/AKT/MTOR (n=5, 29%) and MAPK pathways (n=3,18%); 7 (41%) patients demonstrated ≥1 alterations in tumor suppressor genes (*TP53* in 5 patients), 8 (47%) had impaired epigenetic regulation and DNA methylation, 8 (47%) had aberrant cell cycle regulation, commonly in the cyclin dependent kinases. Ten (59%) patients had alterations in transcriptional regulators. Concurrent mutations affecting cell cycle regulation were noted to occur with aberrant epigenetic regulation (n=6, 35%) and MAPK/PI3K pathway (n=5, 29%).

**Conclusion:**

Every patient had a unique molecular profile with no two patients demonstrating an identical panel of mutations. We identify two emerging novel combinatorial strategies targeting impaired cell cycle arrest, first with epigenetic modifiers and, second, with MAPK/PI3K pathway inhibition.

## INTRODUCTION

Carcinoma of unknown primary (CUP) remains a unique challenge to the clinician in a landscape where algorithms for the diagnosis, management and outcomes of cancers are often histology-dependent. Indeed, CUP represents a heterogeneous group of malignancies with a distinct disease course and biology, often displaying aggressive behavior with a short period of clinical symptoms preceding diagnosis and early dissemination to multiple metastatic sites leading to advanced staging at presentation and [1–3]. CUP is defined as a metastatic cancer without a clearly identified primary site despite an adequate standard workup including an in-depth pathologic analysis with concurrent detailed history, physical examination, and laboratory and radiologic assessments [4].

The preliminary categorization on microscopic evaluation classifi an overarching histology as adenocarcinomas (ranging from well, moderate, poor, or undifferentiated), which represent up to 90% of CUP cases; lesser prevalent characterizations include squamous cell carcinomas, undifferentiated neoplasm, melanoma, sarcoma, or lymphoma seen in the remaining 10% of cases [5, 6]. Next, after identification of preliminary histology, the pathologic evaluation continues with a systematic immunohistochemistry algorithm to identify the general cellular subset, including adenocarcinoma, neuroendocrine, germ-cell, etc. while a further testing based on cytokeratins (CKs) may reveal the detailed phenotypic expression of specifi organ [1].

Progress in gene expression and molecular profiling further aids the sub-classification of the tissue of origin into predominant subtypes of cancer, such as gastrointestinal, gynecologic, etc [7–10]. However, the final definitive histologic identity of the cancer remains elusive. Recent rapid advances in genomic profiling present new opportunities to not only characterize CUP but also offer insight into pathways and cellular systems with aberrations that may be of value as a therapeutic target. To that end, we performed next generation sequencing on patients with advanced, relapsed CUP referred to our phase I clinical trials program to identify potentially future targets for drug development and clinical trial design.

## RESULTS

### Patients characteristics

We identifi 17 consecutive patients with advanced, relapsed CUP on whom adequate tissue was available for molecular profi Patient characteristics are described in Table 1. The median age of this group at the time of diagnosis was 49 years (range, 18-72), while the age at time of initial phase I referral was 54 years (range, 21-75). Patients had a preserved performance status with all having an ECOG PS of 0 (n=2, 12%) or 1 (n=15, 88%). Patients were heavily pretreated with the median number of prior systemic therapies prior to phase I referral being 3 (range, 0-8). On pathologic evaluation, fi of 17 patients (29%) had tumor with a microscopic description of carcinoma, 4 of which was poorly differentiated and one undifferentiated. Four patients' tumors (24%) were characterized as adenocarcinoma, 3 of which were poorly- and one moderately differentiated. Three patients had a squamous cell of unknown primary (two moderately differentiated and one poorly differentiated). Remaining histologies described included moderate to well-differentiated epithelioid neoplasm (n=2, 12%), poorly differentiated neuroendocrine (n=2, 12%), and poorly differentiated sarcomatoid neoplasm (n=1, 6%).

**Table 1:** Characteristics of 17 patients with advanced relapsed CUP seen in the Phase I Clinical Trials Program

Gender	
Male	9 (53%)
Female	8 (47%)
Age at diagnosis, years	
Median	49
Range	18 – 72
Age at phase I referral, years	
Median	54
Range	21-75
ECOG performance status	
0	2 (12%)
1	15 (88%)
2-3	0
No. of prior systemic therapies	
Median	3
Range	0 - 8
Number of molecular aberrations	
Median	2
Range	0 - 8

Abbreviations: ECOG Eastern Cooperative Oncology Group.

### Molecular analysis by histologic subtype

Of the 17 patients who had adequate tissue for molecular profiling, 15 patients (88%) had one or more molecular aberrations identified on the NGS assay; two patients (one squamous cell and one neuroendocrine by histology) did not reveal any identifiable aberrations. The 17 specimen harbored a total of 59 alterations with a median of 2 mutations and a range of 0 to 8. Table 2 details the pathways and cellular process implicated by the identified molecular aberrations. Overall, the most common aberrations led to impaired cell cycle arrest with 8 patients demonstrating 21 aberrations, including 3 patients with *CDKN2A/B* loss and 2 with *CCND1* amplification.

**Table 2:** Cellular processes and pathway with identified aberrations in advanced CUP patients

Processes and pathways implicated	Mutations identified in tested specimen
Apoptosis	MCL1 amplification
Cell cycle regulation	CDKN2A/B loss, SOX2 amplification
	CDK12 Q570*, CCND1 amplification
	FGF19 amplification, FGF3 amplification
	FGF4 amplification, CCNE1 amplification
	CCND1 amplification, SMARCB1 I365fs*22+
Epigenetic regulation	MLL2 R4904*, KDM6A S466
	ARID1A Y1211fs*5, S1929fs*25, and E2250fs*28
	SETD2 G1644*, N2071fs*17
	ATRX R840fs*29, CREBBP S893L
PI3K/AKT/mTOR pathway signaling	PIK3CA H1047R, Q75E, E545K, and amplification
	FBXW7 splice 726+1 G>A, R465H, and W244
MAPK pathway signaling	KRAS amplification, FGFR1 amplification
Transcription regulation	MYCL1 amplification, MYST3 amplification
	EWSR1 EWSR1-CREB1 fusion, EMSY amplification
	SMAD4 P130S, NFE2L2 D11Y
	ETV1 rearrangement, NOTCH1 APIP-NOTCH1 fusion
Tumor suppressor	MDM4 amplification, MDM2 amplification
	TP53 L45P, Q38fs*79, R273C, R248Q, R196*, and R248W
Wnt signaling	CDC73 Q333*, CTNNB1 S33P

We then examined the cellular processes and pathways affected within each histologic subtype (detailed in Table 3) with an emphasis of the two most predominant histology, carcinoma and adenocarcinoma. Of the five patients with a carcinoma, the median number of aberrations was 2 (range 1-8), and the median age at time of phase I evaluation was 65.2 years (range 48.2 – 74.6). Four of the 5 patients demonstrated an aberration leading to activation of the PI3K/AKT/mTOR pathway, including one patient with a *PIK3CA* Q75E mutation and a second patient with both a *PIK3CA* E545K mutation as well as an amplification. Three of these five carcinoma patients demonstrated a mutation in the tumor suppressor *TP53* (R273C, R248Q, R196*) while two patients had five aberrations in cell cycle regulation including *CDKN2A/B* loss, and *CCND1* and *CCNE1* amplifications.

**Table 3:** Mutation profile outcomes by tumor histology

Histology	No. of patients	Median age at diagnosis (years)	Cellular Processes Affected (No. of mutations)
SCC	3	56.5	PI3K/Akt/mTOR pathway (1)
			RTK (1)
			Cell cycle regulation (5)
			Epigenetic regulation (2)
			Wnt signaling (1)
			Tumor suppressor (1)
			Transcription regulation (2)
Adenocarcinoma	4	67.7	Apoptosis (1)
			Cell cycle regulation (3)
			Epigenetic regulation (4)
			PI3K/Akt/mTOR pathway (1)
			RTK-EGFR family (1)
			RTK; angiogenesis regulation (1)
			Transcription regulation (4)
			Tumor suppressor (2)
Sarcomatoid	1	24.7	Transcription regulation
Neuroendocrine	2	19.6	Cell cycle regulation (4)
			Transcription regulation (1)
			Tumor suppressor (1)
			DNA methylation (1)
Epithelioid	2	43.0	Transcription regulation (2)
			Epigenetic regulation (1)
Carcinoma	5	49.2	PI3K/Akt/mTOR pathway (4)
			RAF/MEK/ERK (1)
			Cell cycle regulation (5)
			Tumor suppressor (3)
			Wnt signaling (1)
			RTK (1)

Abbreviations: EGFR epidermal growth factor, RTK receptor tyrosine kinase.

Next, 4 patients had the second predominant histologic subtype of adenocarcinoma. Notably, all 4 patients had an aberration implicated in epigenetic deregulation, specifically in *ARID1A* (Y1211fs*5 and E2250fs*28) in two samples, a dual *SETD2* mutation (G1644*, N2071fs*17) and a *CREBBP* S893L mutation, highlighting a role in targets cellular epigenetics in therapy. Two mutations including *CDKN2A/B* loss leading to impaired cell cycle regulation were also noted.

### Signal transduction mechanisms: Activation of the PI3K and MAPK pathways

Overall 8 (47%) patients had an aberration implication in a signal transduction cascade. Specifically 5 patients (29%) had 6 mutations leading to aberrant activation of the PI3k/Akt/mTOR signaling pathways, including three patients with *PIK3CA* mutations, one in the more common site H1047R, and two in less prevalent sites, Q75E and E545K with a concurrent amplification. Three patients had a mutation with *FBXW7* mutation (one splice 726+1 G>A, a second W244*, and a third R465H). Three patients (n=3, 18%) had aberrant activation of the MAPK pathway with one patient harboring a KRAS amplification and two with *FGFR1* amplification.

### Impaired cell cycle regulation

Overall, 8 (47%) patients had mutations in cell cycle regulators, most commonly in the cyclin dependent kinases. Three patients (18%) harbored a *CDKN2A/B* loss, both genes encode the tumor suppressors p15 and p16, therefore loss of these genes leads to the deregulation of the p16-CDK4/Cyclin/Rb pathway and loss of cell cycle control [11]. Similarly two patients had a *CCND1* amplification, which encodes Cyclin D1, which in turn interacts with the cyclin-dependent kinases Cdk4 and Cdk6, resulting in the phosphorylation and inactivation of Rb and the progression of the cell cycle. Studies have shown that overexpression or amplification of Cyclin D1 may therefore lead to excessive proliferation [12, 13]. Additional aberrations included three (18%) patients with *SOX2* amplification, three (18%) each with amplification in *FGF3, FGF4*, and *FGF19*, two (12%) with *CCND1* amplification, and one with *CCNE1* amplification. *FGF 3, 4* and *19* encode various fibroblast growth factors and are known to be located a region of chromosome 11q13 that also encodes key regulators of cell-cycle progression [12].

### Impaired epigenetic regulation and DNA methylation

Similarly, 8 (47%) had impaired epigenetic regulation and DNA methylation with mutations in most commonly in *ARID1A* which encodes the AT-rich interactive domain-containing protein 1A, a member of the SWI/SNF chromatin remodeling complex. Three different mutations in *ARID1A* were reported (the frameshift mutation Y1211fs*5, S1929fs*25, and E2250fs*28) [14, 15]. Other genes with reported aberrations included *MLL2* R4904* (which is histone methyltransferase that is involved in the transcriptional response to progesterone signaling), *KDM6A* S466 (implicated in the epigenetic regulation of transcription), *SETD2* which encodes a histone lysine-36 methyltransferase (both G1644* and N2071fs*17 in the same patient), *ATRX* R840fs*29 (which encodes a helicase protein and binds tightly to chromatin during chromosomal segregation at mitosis), and *CREBBP* S893L (which encodes proteins acting as intrinsic histone acetyltransferases and as stabilizers within the transcription complex) [16].

### Other aberrations: Tumor suppressors and transcriptional regulators

Seven (41%) patients demonstrated one or more alterations in tumor suppressor genes while 10 (41%) patients had unique alterations in transcriptional regulators. Most commonly, five patients had 6 unique aberrations within *TP53*, specifically R273C, R248Q, R 196*, and R248W including one patient with an adenocarcinoma who had a dual mutation within *TP53* with a relatively uncommon N-terminal missense mutation L45P and a concurrent Q38fs*79, which is frameshift mutation leading to the truncation of the p53 protein prior to the conserved DNA-binding domain region. This patient's tumor demonstrated 8 mutations overall including alterations in the PI3K, MAPK pathways (mutations in *FBXW7*, *FGFR1*) with concurrent epigenetic and transcriptional deregulation, specifically *ARID1A*, *ETV1* rearrangement, *NOTCH1 APIP-NOTCH1* fusion, and *MYST3* amplification. One patient had an *MDM2* amplification while a second showed an *MDM4* amplification, both of which, when amplified, act as tumor suppressors acts to inhibit the activity of p53 [17, 18].

### Concurrent mutations: Deregulation of cell cycle with PI3K pathway activation

**Figure 1:**
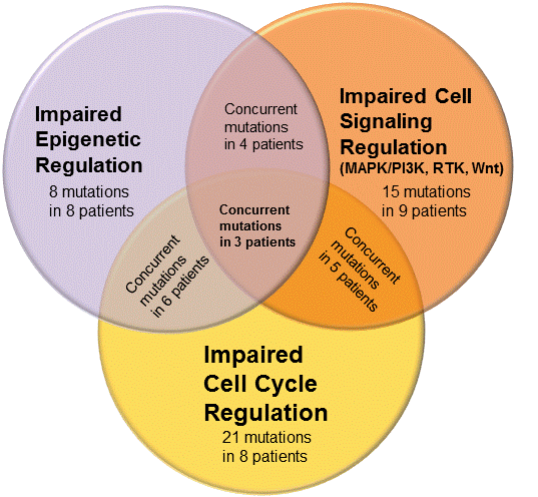
Areas of dysregulation identified on molecular profiling of CUP.

Of the 17 patients, we further analyzed the incidence of concurrent mutations affecting two or more pathways and cell processes (Figure 1). Of the 8 patients with mutations leading to deregulation of the cell cycle, we identified 4 (50%) patients with a concurrent mutation in the PI3K cascade and one with *ERBB2* amplification, part of the epidermal growth factor receptor (EGFR) family. Similarly, of the 5 patients with mutations linked to the PI3K pathway, 4 (80%) occurred concurrently with an aberrance in cell cycle regulation (including *CDK12*, *CCNE1* amplification in 2 patients, *CDKN2A/B* loss). Furthermore, of the 8 patients with cell cycle aberrations, six (75%) had a concurrent mutations associated with epigenetic regulation and DNA methylation (including mutations in *KDM6A*, *MLL2*, *ARID1A* in 2 patients, and *SETD2*). Outcomes of molecular profiling for each patient are detailed in Table 4.

**Table 4:** Molecular profile outcomes for all CUP patients

Patient	Age at diagnosis (years)	No. of prior systemic therapies	Histology	No. of mutants	Specific mutations and aberrations identified	
1	21.0	2	Epithelioid	1	EWSR1-ATF1 fusion	
2	59.0	2	Squamous cell	6	PIK3CA H1047R	KDM6A S466*
					ALK L560F	CDC73 Q333*
					SOX2 amplification	CDK12 Q570*
3	17.9	2	Neuroendocrine	7	CCND1 amplification	FGF19 amplification
					MYCL1 amplification	FGF3 amplification
					MDM4 amplification	FGF4 amplification
					MLL2 R4904*	
4	21.2	3	Neuroendocrine	0	N/A	
5	33.8	1	Adenocarcinoma	8	FBXW7 splice 726+1 G>A	APIP-NOTCH1 fusion
					FGFR1 amplification	TP53 L45P, Q38fs*79
					ARID1A Y1211fs*5	MYST3 amplification
					ETV1 rearrangement	
6	49.4	5	Squamous cell	0	N/A	
7	70.2	8	Carcinoma	4	FBXW7 R465H	CCNE1 amplification
					PIK3CA Q75E	TP53 R273C
8	72.0	6	Carcinoma	2	FBXW7 W244*	TP53 R248Q
9	49.2	2	Carcinoma	8	PIK3CA E545K & amplification	FGFR1 amplification
						SOX2 amplification
					KRAS amplification	TP53 R196*
					CCND1 amplification	CDKN2A/B loss
10	44.0	3	Carcinoma	1	CTNNB1 S33P	
11	72.4	3	Adenocarcinoma	2	SMARCB1 I365fs*22+	SETD2 G1644*, N2071fs*17
12	24.7	0	Sarcomatoid	1	EWSR1-CREB1 fusion	
13	65.0	3	Epithelioid	2	EWSR1-NR4A3 fusion	ATRX R840fs*29
14	56.5	2	Squamous cell	7	BRCA2 W1692fs*3	ARID1A S1929fs*25
					CDKN2A/B loss	EMSY amplification
					MDM2 amplification	SMAD4 P130S
					SOX2 amplification	
15	63.8	7	Adenocarcinoma	5	ERBB2 amplification	TP53 R248W
					CDKN2A/B loss	CREBBP S893L
					MCL1 amplification	
16	71.7	8	Adenocarcinoma	3	FGFR3 R399C	NFE2L2 D11Y
					ARID1A E2250fs*28	
17	48.0	1	Carcinoma	2	NF2 splice site 448-1G>A	

### Clinical outcomes

Of the 17 patients, 11 (65%) elected to participate in a phase I clinical trial after having met all eligibility criteria. The remaining 6 proceeded to receive conventional chemotherapies or a clinical trial closer to home. Table 5 details the specific clinical trials and outcomes of each patient in our study. One patient received monotherapy while the remaining 10 received a combination of 2 or more drugs. All 11 patients were treated with a regimen that included a targeted therapy agent. Of the 11 patients treated on phase I clinical trials, over half (n=7, 64%) received a therapy matched with their mutational profile. The best tumor responses noted were stable disease lasting 4 or more months seen in 4 patients, 3 of whom had therapy matched to their mutational profile. Two of the 11 treated patients currently remain on therapy for over 8 months, first on a combination of carboplatin, bevacizumab, and temsirolimus and the second on crizotinib and pazopanib.

**Table 5:** Clinical outcomes of CUP patients treatment on a phase I therapy

Pt #	Age/Sex	Phase I Regimen	Therapy matched to molecular aberration	Best Response per RECIST	Phase I PFS (m)
1	22/M	Not treated*	-	-	-
2	60/F	A novel PI3K inhibitor	Yes	SD	5.9
3	21/M	Vandetanib + everolimus	No	PD overall (SD of target lesions, but developed a new liver lesion)	2.0
4	25/F	Not treated*	-	-	-
5	36/F	Carboplatin + bevacizumab + temsirolimus	Yes	SD (On study)	8.0+
6	54/M	Not treated*	-	-	-
7	75/F	Everolimus + anastrozole	Yes	PD	3.3
8	74/M	Sirolimus + hydroxychloroquine	Yes	SD	4.5
9	50/M	Lenalidomide + temsirolimus	Yes	PD	1.4
10	51/F	Not treated*	-	-	-
11	73/M	Not treated*	-	-	-
12	28/F	Crizotinib + pazopanib	No	SD (On study)	8.9+
13	67/F	Vandetanib + everolimus	No	PD	2.7
14	57/F	Liposomal doxorubicin + bevacizumab + temsirolimus	Yes	PD overall (SD of lung lesions, PD of liver lesion)	2.8
15	65/M	Not treated*	-	-	-
16	74/M	Everolimus + denosumab	No	PD	1.5
17	48/M	Lapatinib + sirolimus	Yes	PD (mixed response)	1.7

* Not treated on a phase I trial due to decision to pursue alternate therapies.

Abbreviations: PD progressive disease, PI3K phosphatidylinositol 3-kinase, PFS progression free survival, RECIST Response Evaluation Criteria In Solid Tumors, SD stable disease.

## DISCUSSION

Our exploratory analysis provides insight into the molecular fingerprint of advanced, relapsed carcinoma of unknown primary. Indeed, no two patients had an identical molecular profile, highlighting the need for truly personalized therapy for this patient population with few effective therapeutic options particularly in the relapsed setting.

The role for molecular profiling to identify the tissue of origin has been explored in depth in CUP; however data into identification of somatic alterations as identifiers of tissue of origin continues to be slowly developed where coexistence patterns in concurrent mutations may suggest the organ system origin. Existing large scale molecular profiling analysis notes that *PIK3CA* H1047R and *KRAS* mutations appear to coexist in genitourinary cancers and breast cancers when noted, and are less prevalent concurrently in colorectal cancer [19].

Our early analysis suggests emerging patterns of aberrations where impaired cell cycle arrest has been observed concurrently with either epigenetic deregulation or activation of the cell signaling. Aberrations in signal transduction pathways including the PI3K and MAPK pathways were consistently observed. Specific mutations including *FBXW7* have been implicated in the activation of *PI3K/Akt/mTOR* signaling pathways. This mutation has been identified in a variety of solid tumors including colorectal cancer (14%), squamous cell cancer of head and neck (11%), liver (8%), and ovarian cancers (3%) [20]. These *FBXW7* mutations have been showed to occur prior to the F-box domain and the highly conserved WD40 repeat region, an area targeted by proteasome-mediated degradation processes. Therefore, mutations in this region have implicated in the stabilization of oncogenic intermediates including the mTOR signaling protein. Early preclinical data has revealed response to the mTOR inhibitor rapamycin in cell lines with the inactivation of Fbxw7 and confers sensitivity to rapamycin, an mTOR inhibitor, suggesting a potential role for the more widely used mTOR inhibitors temsirolimus and everolimus; however their sensitivities to rapalogs remain to be studied prospectively as retrospective reviews of patient outcomes have not shown a definitive signal of activity [20, 21]. Furthermore, early data also suggests that inactivation of Fbxw7 may confer a resistance to anti-tubulin chemotherapies [22]. Similarly patients harboring deregulation of the PI3K pathway through *PIK3CA* mutations including H1047R have been shown to have a response rate (defined as stable disease ≥6 months or partial response) of 45% in patients with all advanced cancers when treated with a PI3K pathway inhibitor (including mTOR inhibitors) [19].

Our analysis also highlights the emerging description of epigenetic deregulation in advanced CUP patients. In a subset of patients, the identification of somatic mutations in epigenetic genes encoding proteins which lead to alterations in specific DNA methylation and chromatin modification patterns, most commonly through inappropriate gene silencing [23, 24]. The increased prevalence of somatic mutations noted in CUP patients that lead to epigenetic changes reinforces the role for studying classes of drugs, specifically the histone deacetylase inhibitors such as vorinostat and panobinostat in this patient population, particularly in combination.

Furthermore, the observation of concurrent mutations further hopes to identify combinatorial strategies for this malignancy. In our set of patients, 4 of 5 (80%) patients with a *PIK3CA* mutation had a concurrent aberrance in cell cycle regulation. While 6 of 8 (75%) patients with a cell cycle aberration had a coexisting mutation associated with epigenetic regulation and DNA methylation, highlighting two potential combinations to be explored with therapeutic intent. Additionally histology-specific subset analysis reveals early insight into pathways specifically deregulated in our larger subset of patients, specifically the patient with poorly differentiated carcinoma and adenocarcinoma. In the carcinoma subset, 4 of five patients demonstrated an aberration leading to activation of the PI3K/AKT/mTOR pathway, suggesting that activation of this cascade was more closely associated with this histologic subset when compared to the other 4 subsets. In deed all 4 samples of adenocarcinoma, which was the second most predominant subtype among our CUP patients, demonstrated an aberration implicated in epigenetic deregulation.

Our preliminary analysis highlights areas of future study but also does raise limitations. Clinical outcomes have yet to be explored longitudinally, particularly given that efforts were made to match each patient to a clinical trial with evidence of target inhibition based on their mutation profi However the primary limitations of this effort were availability of specifi clinical trials at time of patient need as well as time and travel commitments to meet the clinical trial participation requirements. Moreover, there exists a selection bias and its associated implications on tumor biology given that the molecular profi was completed only on patients who were well enough to be considered for a referral to a phase I clinical trial, leading to a small sample size with tumor histologic heterogeneity. Nonetheless, these early patterns reveal cellular processes that are deregulated concurrent with signaling mechanisms and epigenetic modulations and represent novel combination therapies to be pursued in preclinical models and ultimately early phase clinical trials.

## MATERIALS AND METHODS

### Patient selection and treatment

We identified consecutive patients with CUP referred to the Clinical Center for Targeted Therapy (Phase I Clinical Trials Program) at MD Anderson Cancer Center starting from January 1, 2011 to December 31, 2013. Eligibility criteria for participation in phase I clinical trials included age >18 years, presence of metastatic or unresectable disease, measurable disease per Response Evaluation Criteria in Solid Tumors (RECIST) 1.0, Eastern Cooperative Oncology Group (ECOG) performance status (PS) of 0-1, and a life expectancy >3 months. Premenopausal women were required to have a negative pregnancy test and patients of childbearing potential to use contraception. Further eligibility criteria varied according to the particular study and all patients gave informed consent. All clinical trials were approved by the MD Anderson Institutional Review Board. Descriptive statistics summarized the patients' characteristics.

### Pathologic evaluation and mutational analyses and detection

Original hematoxylin and eosin slides were reviewed by an institutional pathologist to confirm CUP. Additional immunohistochemical staining to assist in the identification of tissue of origin was conducted as per pathologist's discretion. Archival formalin-fixed paraffin-embedded (FFPE) slides were then obtained and cut into 10 separate 5-mm sections. Next-generation sequencing from FFPE sections was completed in a Clinical Laboratory Improvement Amendments (CLIA)-certified laboratory using the Illumina HiSeq2000 platform (Foundation Medicine, Cambridge, MA, USA). Over 3230 exons of 236 cancer-related genes, plus over 47 introns from 19 genes often rearranged in cancer were fully sequenced for point mutations, insertions/deletions, copy number alterations (CNAs), select gene fusions, and variants of unknown significance. Then, the aberrations were classified into 10 encompassing categories based on the pathway or cellular processes where they have been implicated, i.e. aberrations that affect apoptosis, cell cycle regulation, epigenetic modulation/DNA methylation, signal transduction mechanism (PI3K, MAPK, Wnt or a receptor tyrosine kinase[RTK]), transcription regulation, or tumor suppressor.
